# The Importance of Extensional Rheology in Bolus Control during Swallowing

**DOI:** 10.1038/s41598-019-52269-4

**Published:** 2019-11-06

**Authors:** Enrico Karsten Hadde, Julie Ann Yvette Cichero, Shaofeng Zhao, Wei Chen, Jianshe Chen

**Affiliations:** 1 School of Food Science and Biotechnology, Zhejiang Gongshang University, Hangzhou, China; 2 School of Pharmacy, The University of Queensland, Brisbane, Australia; 3 The 1st Affiliated Hospital of Soochow University, Suzhou, China; 4 Present Address: The Eight Affiliated Hospital, Sun Yat-sen University, Shenzhen, China

**Keywords:** Rheology, Oral manifestations

## Abstract

Thickened fluids are commonly used in the medical management of individuals who suffer swallowing difficulty (known as dysphagia). Previous studies have shown that the rheological properties of a liquid affect the flow behavior of the bolus in swallowing, such as pharyngeal transit time. While there is no doubt that shear rheology is a highly important factor for bolus flow, it is suspected that extensional properties of a liquid bolus also plays an important role in swallowing, due to elongation of the bolus as it flows through the oropharynx. Our aim in this work was to observe the effect of extensional viscosity on pharyngeal transit time and elongation of the bolus during swallowing. Eight samples of thickened liquid barium that were shear-controlled, but varied in extensional viscosity and two samples that were extensional-controlled, but varied in shear viscosity were swallowed by eight healthy individuals. Data were collected under lateral view of videofluoroscopy swallow study (VFSS); measures of pharyngeal transit time and the ratio of the length to the width of the bolus on the frame of Upper Esophageal Sphincter (UES) opening were taken from the VFSS recordings. It was observed that the pharyngeal transit time generally increases when the fluids are thickened to higher IDDSI consistency. Additionally, higher extensional viscosity fluids reduced the elongation of the bolus during swallowing, thus potentially reducing the risk of post-swallow residue due to bolus breakage. This study confirmed the relevance of the extensional viscosity of the bolus in swallowing.

## Introduction

Difficulty of swallowing food or fluid, known as dysphagia is a major issue throughout the world^1,2^. Consequences of dysphagia leads to reduced food intake which may in turn cause malnutrition, and potentially aspiration pneumonia and asphyxiation^3,4^. Dysphagia is a common phenomenon to elderly populations all over the world^5,6^. Texture modifications of foods and thickened fluids has been proven to be a valid form to promote safe and efficient swallowing for dysphagia patients^1,7,8^. It has been reported that prescription of thickened fluids has become the most recommended treatment made by clinicians^9^. In particular, thickened fluids can efficiently increase the duration of swallowing for pharyngeal transit time measures, thus allowing better oral and pharyngeal coordination and enhancement of safe swallowing^7,10,11^. However, it is not always easy to obtain the correct consistency of thickened fluids. The patient may face serious consequences if the prescription is not followed^4,12^.

In 2013, International Dysphagia Diet Standardisation Initiative (IDDSI), a group of volunteers from diverse professions was established to develop a global standardized terminology and definitions for texture-modified foods and thickened fluids for individuals with dysphagia^13^. According to this global standard, fluids are divided into 5 grades from Level 0–4, while foods are divided into 5 grades from 3–7^13^. Previous studies had shown that understanding the rheological properties of thickened fluid is advantageous in designing better-controlled fluids^14,15^. For example, it was reported that higher shear viscosity slowed down the flow of the bolus in the pharynx, thus leading to longer pharyngeal transit time. A recent review by Nishinari *et al*.^16^ reported that fluid cohesiveness plays an important role for safe swallowing for individuals with dysphagia. Cohesiveness is defined as the strength of internal bonds making up the body of the product^17^. Less cohesive fluids have a risk to fracture to multiple droplets, allowing the bolus to flow into the oesophagus, as well as the airway which may cause aspiration. Additionally, there is a risk that some of the bolus may get caught in the pharynx as residue, requiring multiple swallows for the individuals to clear the residue. This residue may also be inhaled into the airways when breathing resumes after swallowing^18^.

It is hypothesised that the extensional viscosity as well as shear viscosity of the fluid affect bolus flow. It has been reported that during swallowing the bolus is elongated and subjected to extensional stress^19,20^. Previous studies showed that for similar shear viscosity at 50 s^−1^, the extensional viscosity of the fluids was highly dependent on the type of thickeners^21–23^. For example, fluids that were thickened with xanthan gum thickener have much higher maximum extensional viscosity than fluids that were thickened with modified starch thickener at similar shear viscosity at 50 s^−1^. Mackley *et al*.^23^ demonstrated using a twin piston filament stretching device (Cambridge Trimaster) that starch based thickeners stretched in a non-homogenous fashion leading to premature breakage of the bolus. In contrast, xanthan gum thickener samples showed uniform deformation and much longer lifetime thread decay. The microstructure of the thickeners provides some explanation for this observation. When viewed under an optical micrograph, the starch thickened samples were heterogeneous showing distinct granules, whereas the xanthan gum thickened sample were homogeneous. Additionally, Hadde *et al*.^24^ reported that the extensional viscosity of thickened skim milk is lower than thickened water for a given amount of added thickener because of lower surface tension of thickened skim milk compared to thickened water. Therefore, the extensional viscosity of the fluid may be very different even though both fluids were thickened to similar shear viscosity. It is hypothesised that the maximum extensional viscosity of the fluid is related to the cohesiveness of the fluid^21^. Nishinari *et al*.^16^ reported that fluid cohesiveness is highly correlated with the extensibility of the fluid. In addition, the significance of bolus’ extensional viscosity in swallowing is still not fully understood. Clinical studies are required to evaluate the relevance of extensional viscosity in swallowing. The objective of current study was to:

- Observe the effect of extensional viscosity on the bolus pharyngeal transit time and the elongation of the bolus during swallowing,
- Evaluate the flow behavior of thickened fluids during swallowing through Videofluoroscopy Swallow Study (VFSS),

Healthy individuals were recruited in this experiment to obtain normative data. It is noted that the effects of bolus properties to swallowing behaviour will differ between dysphagia patients and healthy individuals.

## Materials and Methods

### Participants

Eight healthy individuals (6 males and 2 female, age range = 22–30 years) were recruited for this study. The participants were university students based in Hangzhou, China. All participants were non-smokers with average body weight and did not have any abnormalities or treatment of surgery to the head and neck before or during data collection. Informed consent was obtained prior to commencement of the experiment. This project was approved by the ethics committee of the School of Food Science and Biotechnology, Zhejiang Gongshang University (No. 2018011601). All methods were performed in accordance with the relevant guidelines and regulations.

### Sample preparation

The liquid used in this study was still mineral water (Nongfu Spring, Hangzhou, China). Three commercial thickener products were selected: Resource ThickenUp^®^Clear^TM^ (RTC), Nestlé (xanthan gum, maltodextrin), Hehongchun, QingDao Siboran (xanthan gum and potato starch) and ThickenUp, Nestlé (modified maize starch). Barium sulphate was used due to its opaqueness, allowing the fluid to be monitored during the VFSS. The barium sulphate products used were supplied by the hospital in Suzhou (The First Affiliated Hospital of Soochow University) which is commonly used for VFSS in China. Barium sulphate powder of 60 grams was mixed with 100 mL of water and stirred until the powder was evenly dispersed. This barium concentration recipe was recommended by the clinicians in the hospital to make the liquid radiopaque for the videofluoroscopy observation. The desired quantity of thickener was added into an empty container and the barium sulphate solution was added. The mixture was stirred vigorously with a spoon until the thickener was completely dissolved. The samples were then allowed to stand for 30 minutes for appropriate hydration^25^. Ten samples of thickened barium with varying thickeners and varying amount of thickeners were prepared (See Table 1). For each sample, three repeats of swallow were conducted for repeatability, therefore a total of thirty samples were swallowed by each participant over three sessions. All of the samples were re-stirred after standing before they were prepared in syringes labelled with three random digit identifiers so that the participants did not know the identity of the solutions.

**Table 1:** Summary of the rheological parameters of the samples (±95% confidence interval).

Sample No.	Thickener	Thickener Concentration %	Shear Viscosity at 50 s^−1^ (mPa.s)	Max. Extensional Viscosity (Pa.s)	IDDSI Level Leftover volume (mL)
1	RTC	0.71	76 ± 6	1.175 ± 0.044	IDDSI Level 1 3.82 ± 0.11
2	ThickenUp	3.62	69 ± 19	Too low	IDDSI Level 1 2.22 ± 0.34
3	RTC	1.18	116 ± 1	1.985 ± 0.116	IDDSI Level 2 4.62 ± 0.15
4	ThickenUp	4.35	125 ± 11	Too low	IDDSI Level 2 7.33 ± 0.47
5	RTC	2.61	301 ± 2	5.925 ± 0.354	IDDSI Level 3 9.22 ± 0.13
6	ThickenUp	4.86	328 ± 30	Too low	IDDSI Level 3 8.82 ± 0.16
7	RTC	5.60	724 ± 32	34.58 ± 5.448	IDDSI Level 4 (fork drip test)
8	ThickenUp	5.34	725 ± 68	0.410 ± 0.020	IDDSI Level 4 (fork drip test)
9	Hehongchun	4.97	529 ± 18	5.842 ± 0.759	IDDSI Level 3 9.76 ± 0.05
10	Hehongchun	9.21	1222 ± 102	24.41 ± 3.302	IDDSI Level 4 (fork drip test)

### Rheological characterization

The shear viscosity of the samples was measured on a Discovery HR-2 (TA Instruments Ltd., U.S.). Cone and plate geometry was selected; diameter: 40 mm; angle: 2°0′29″. A total of 0.6 ml of fluid sample was used for each test measurement. It was observed that some of the barium sulphate was insoluble in sample 2 and sample 4. To reduce the particle settling effect in the measurement, double wall concentric cylinder geometry was selected for this particular samples (cup: outer diameter 37.03 mm, inner diameter 30.20 mm; bob: outer diameter 34.99 mm, inner diameter 32.01 mm. A total of 11 ml of fluid sample was used for each test measurement. The rheometer was operated in strain-controlled mode where the shear rate was specified and the required torque (shear stress) was recorded. Steady shear sweep tests were performed to measure the apparent shear viscosity of the fluids from 1 to 100 s^−1^ and the shear viscosity at 50 s^−1^ was noted. The measurements were repeated in duplicate with different samples used. All testing was conducted at room temperature (25 °C).

The maximum extensional viscosity of the samples was measured using commercial filament break-up equipment (Haake CaBER1 extensional rheometer, ThermoHaake, GmbH, Karlsruhe, Germany). The experimental methodology to measure the extensional viscosity of the samples was based on the model presented by Hadde and Chen^21^. The principle of the method is to measure the diameter of a filament stretched between two plate and the thinning at the mid-point of a filament. The two plates have a diameter of 6 mm with a 3 mm initial gap. The sample was then stretched linearly for 50 milliseconds to a final gap of 10 mm. The diameter of the filament was measured continuously at the mid-point of the final gap height (*D*_*mid*_) with the help of a laser beam. The filament stretching and subsequent filament thinning behavior of the samples was also captured by a high speed camera (PCO dimax HD). The camera was set to capture the image at 1000 fps with a shutter time of 1 µs. The measurements were repeated ten times for one sample and the mean of the ten measured was calculated for the analysis. All testing was conducted at room temperature (25 °C). The apparent extensional viscosity *η*_*E*_ can be calculated from the filament diameter thinning behavior, when the top plate reaches the final gap, using an equation developed by McKinley and Tripathi^26^:

1 $${\eta }_{E}=(2X-1)\frac{\sigma }{-\frac{d{D}_{mid}(t)}{dt}},$$

where σ is the surface tension of the fluid and *X* is a geometry coefficient that takes into account the shape of the filament deviation during thinning due to inertia and gravity^26,27^. It was reported that *X* = 0.7127 was the most suitable value for highly viscous fluids^26^.

To observe the relevance of shear viscosity and extensional viscosity to swallowing function and swallowing safety, the samples were thickened to a shear viscosity that is clinically relevant for people with dysphagia. The samples were prepared in a way that the samples have similar shear viscosity at 50 s^−1^, but different maximum extensional viscosity. Samples that were thickened with RTC have higher maximum extensional viscosity than samples that were thickened with ThickenUp at similar shear viscosity at 50 s^−1^. The samples were thickened to IDDSI Level 1–4. IDDSI flow test was conducted to determine the IDDSI level 1 – level 3. 10 mL slip tip syringe (BD^TM^ syringes, manufacturer code 301604) was used to measure the IDDSI levels of the samples. Prior to the test, the outlet of the syringe was sealed by covering the nozzle of the syringe with the finger and the samples were filled up to 10 mL line. The flow test was started by removing the finger from the nozzle and allowing the sample to flow out of the syringe for 10 seconds. The outlet of the syringe was then sealed again with the finger and the remaining volume of the sample in the syringe was recorded. The samples are then categorized based on the remaining volume (e.g. remaining volume < 1 mL is Level 0, remaining volume between 1–4 mL is Level 1, remaining volume between 4–8 mL is Level 2 and remaining volume > 8 mL is Level 3)^13^. IDDSI Fork Drip test was conducted to determine the IDDSI level 4. A standard plastic fork was used to conduct the IDDSI Fork Drip test by assessing whether the samples flow through the prongs of a fork and comparing against the detailed descriptions of each level^4,28^. Additionally, samples 9 and 10 were thickened with Hehongchun thickener, were selected to be compared with RTC samples (e.g. samples 5 and 9 and samples 7 and 10), so that the samples have similar maximum extensional viscosity, but different shear viscosity at 50 s^−1^.

### Procedure

The participants were briefed about the aim of the project prior to participating the experiment and completed a consent form. The participants were required to visit a hospital in Suzhou, China for three times. In the first visit, three samples (Sample 7, 8 & 10) and the repeats were swallowed (i.e. a total of nine swallows) by the participants. Subsequently, four samples (Sample 1, 2, 3 & 4) and the repeats were swallowed (i.e. twelve swallows) by the participants on their second visit. Finally, three samples (Sample 5, 6 & 9) and the repeats were swallowed (i.e. nine swallows) by the participants on their last visit. The participants were aware that three repeats were required for each sample, however they were not able to distinguish the samples as the samples were presented to the participants in a random order with similar appearance. The participants were asked to drink mineral water (Nongfu Spring, Hangzhou, China) provided before each swallowing test. 10 mL of samples were then placed into the participant’s mouth using a syringe and were told not to swallow the sample until instructed to do so by the Radiologist. The participants were instructed to swallow the sample with one ‘swallow’. Appropriate financial award was given to each participant to compensate their time and effort.

**Figure 1:**
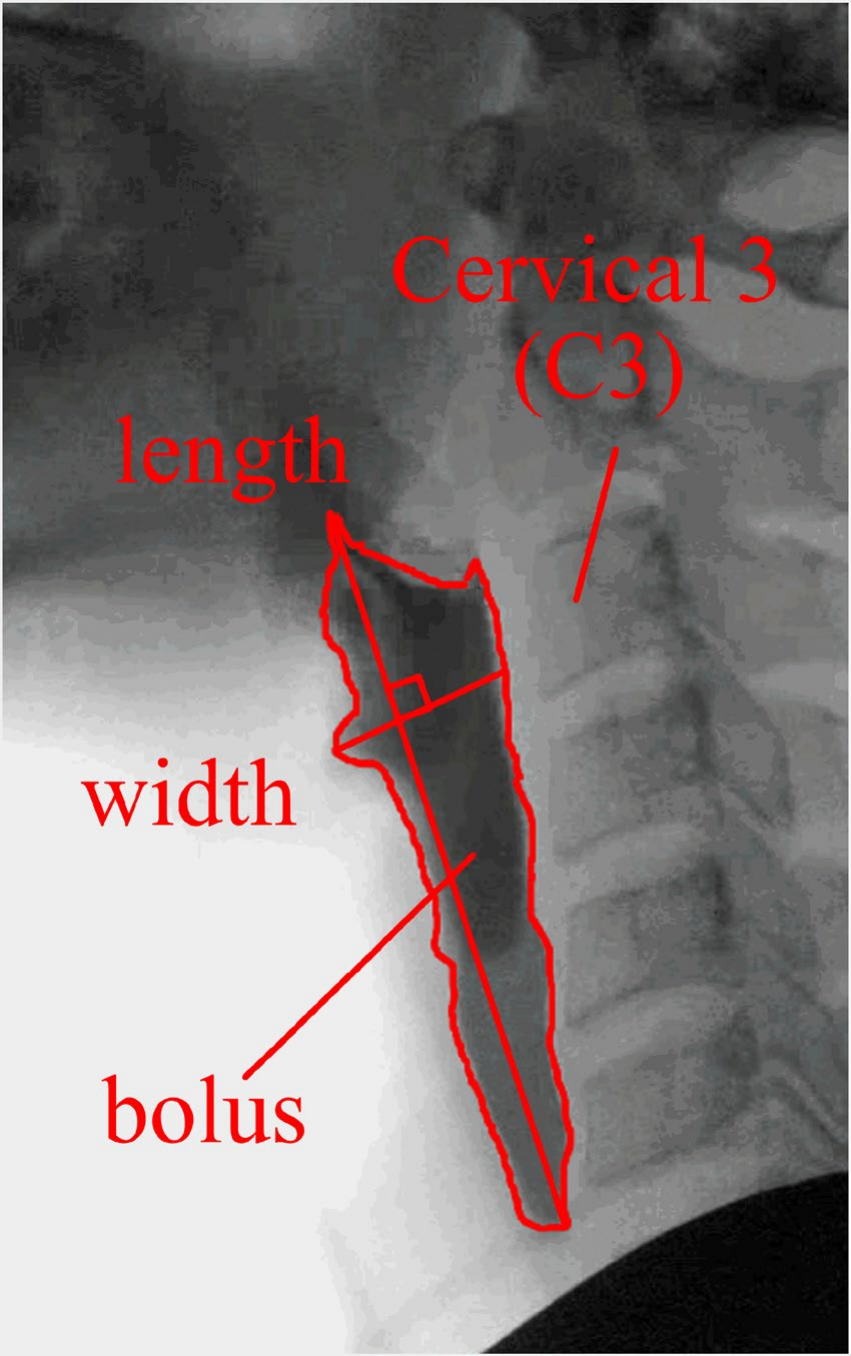
Photographic of the aspect ratio of the bolus at the UES opening. Aspect ratio = 4.2 (length/width).

The videofluoroscopy tests were conducted in hospital set up by clinicians and under supervision of medical doctors. The data was stored in the windows media video format (wmv) with frame rate of 25 fps. Two parameters were extracted from the swallowing video: pharyngeal transit time and aspect ratio of the bolus on the frame of Upper Esophageal Sphincter (UES) opening. ImageJ, a Java-based image processing program^29^ was used to extract the parameters. The video captured was converted into pictures for every 40 milliseconds. The pharyngeal transit time was defined as the time from when the bolus tail is at the oral pillars of fauces (near the oral tonsils) to when it passes through the UES (i.e. bolus tail leaves the pharynx)^30^. The aspect ratio of the bolus at the UES opening was the ratio of the bolus length from bolus tail to bolus head to its width at Cervical 3 (C3) (length/width) (illustrated in Fig. 1).

### Data analysis and statistical methods

For each of the samples, the mean and 95% confidence limits of pharyngeal transit time and aspect ratio of the bolus were calculated. An analysis of variance (ANOVA), with Tukey’s method to make pairwise comparisons, was carried out to determine whether there were any statistically significant differences. A p-value below 0.05 was regarded as statistically significant. The statistical analysis was performed using Minitab 17 Statistical Software.

### Ethical statements

Ethical review: This research was conducted with the approval of research ethical committee at the School of Food Science and Biotechnology, Zhejiang Gongshang University (No. 2018011601)

Informed consent: Informed consent was obtained from all participants before inclusion in this study.

## Results

### Rheological characterization

**Figure 2:**
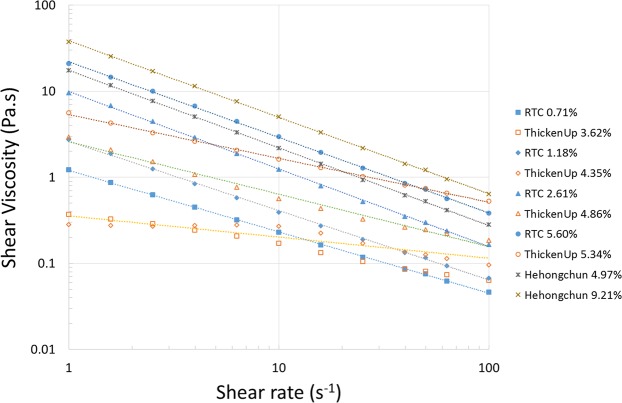
Shear viscosity profile of all thickened barium samples over two orders of magnitudes of shear rate.

**Figure 3:**
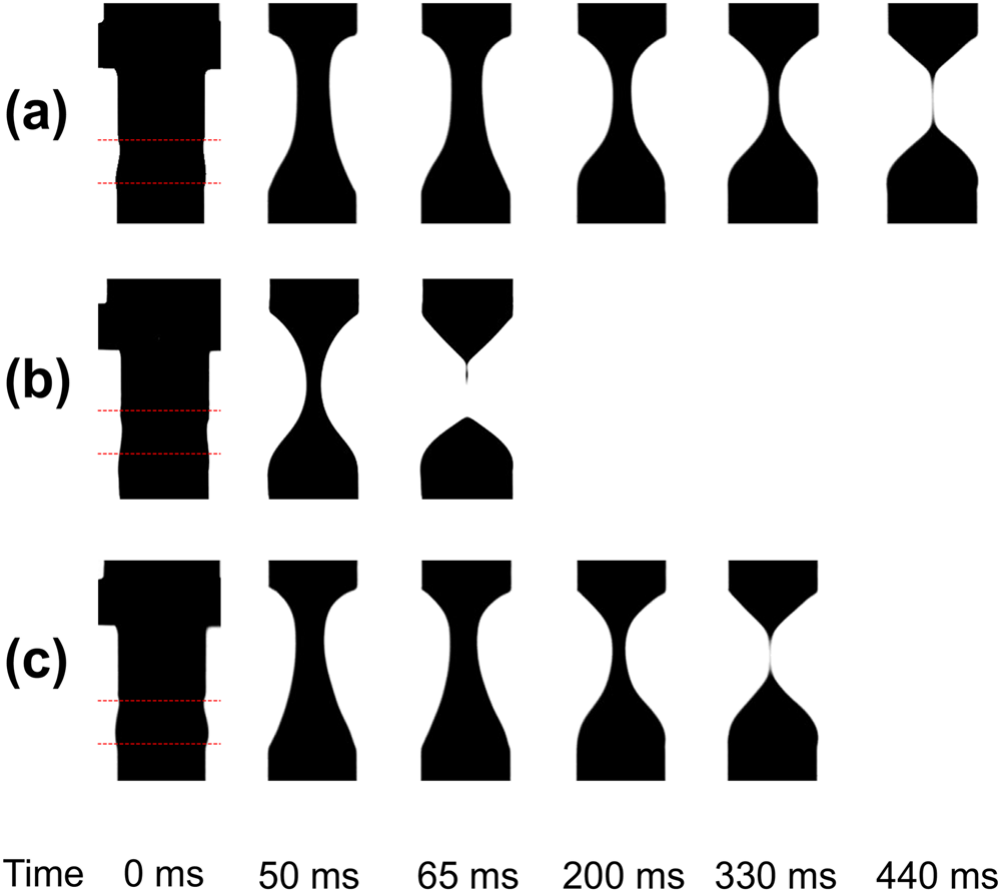
Photographic sequences of the filament break-up captured with CaBER for thickened barium for (**a**) sample 5 (2.61% RTC), (**b**) sample 6 (4.86% ThickenUp), (**c**) sample 9 (4.97% Hehongchun). Initial gap 3 mm, final gap 10 mm, piston diameter 6 mm. The piston stopped moving at 50 ms.

Figure 2 shows the shear viscosity profile of thickened barium samples over two orders of magnitudes of shear rate. It can be seen that all of the samples tested exhibited a shear-thinning behaviour and best followed a power law model as the shear rate increased. Figure 3 shows the photographic sequences of the filament break-up captured with CaBER for thickened barium for sample 5 (2.61% RTC), sample 6 (4.86% ThickenUp) and sample 9 (4.97% Hehongchun). These samples were selected because sample 5 and sample 6 were shear-controlled, but varied extensional viscosity, while sample 5 and sample 9 were extensional-controlled, but varied shear viscosity. It should be noted that some of the samples that were thickened with ThickenUp displayed filament breakage before the piston reached the final gap, thus the maximum extensional viscosity could not be determined. This is because the extensional viscosity of the samples were very low. The summary of the rheological parameters of the samples can be seen in Table 1.

### Videofluoroscopy swallow study (VFSS)

**Figure 4:**
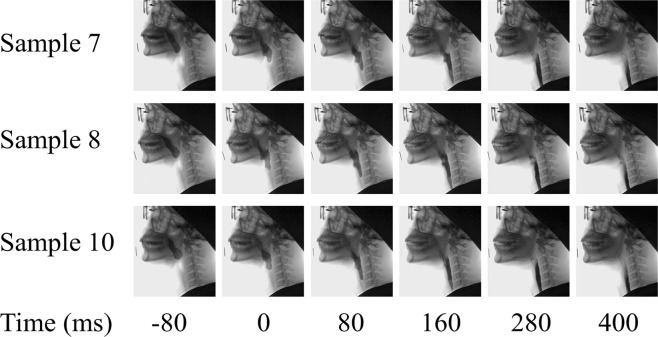
Photographic sequences of the swallowing action for sample 7 (shear viscosity at 50 s^−1^ = 0.724 Pa.s and maximum extensional viscosity = 34.58 Pa.s), sample 8 (shear viscosity at 50 s^−1^ = 0.725 Pa.s and maximum extensional viscosity = 0.41 Pa.s) and sample 10 (shear viscosity at 50 s^−1^ = 1.222 Pa.s and maximum extensional viscosity = 24.41 Pa.s).

Figure 4 shows the photographic sequence of the swallowing action for sample 7 (shear viscosity at 50 s^−1^ = 0.724 Pa.s and maximum extensional viscosity = 34.58 Pa.s), sample 8 (shear viscosity at 50 s^−1^ = 0.725 Pa.s and maximum extensional viscosity = 0.41 Pa.s) and sample 10 (shear viscosity at 50 s^−1^ = 1.222 Pa.s and maximum extensional viscosity = 24.41 Pa.s) swallowed by the same person. As per the radiologist, penetration-aspiration was not observed for any of the participants on any of the samples. It was observed that the rate of the bolus flow was similar for all of the samples. Additionally, a slight residual coating was observed in the pharyngeal area of the participants after swallowing sample 8, but none was observed for sample 7 and 10.

**Figure 5:**
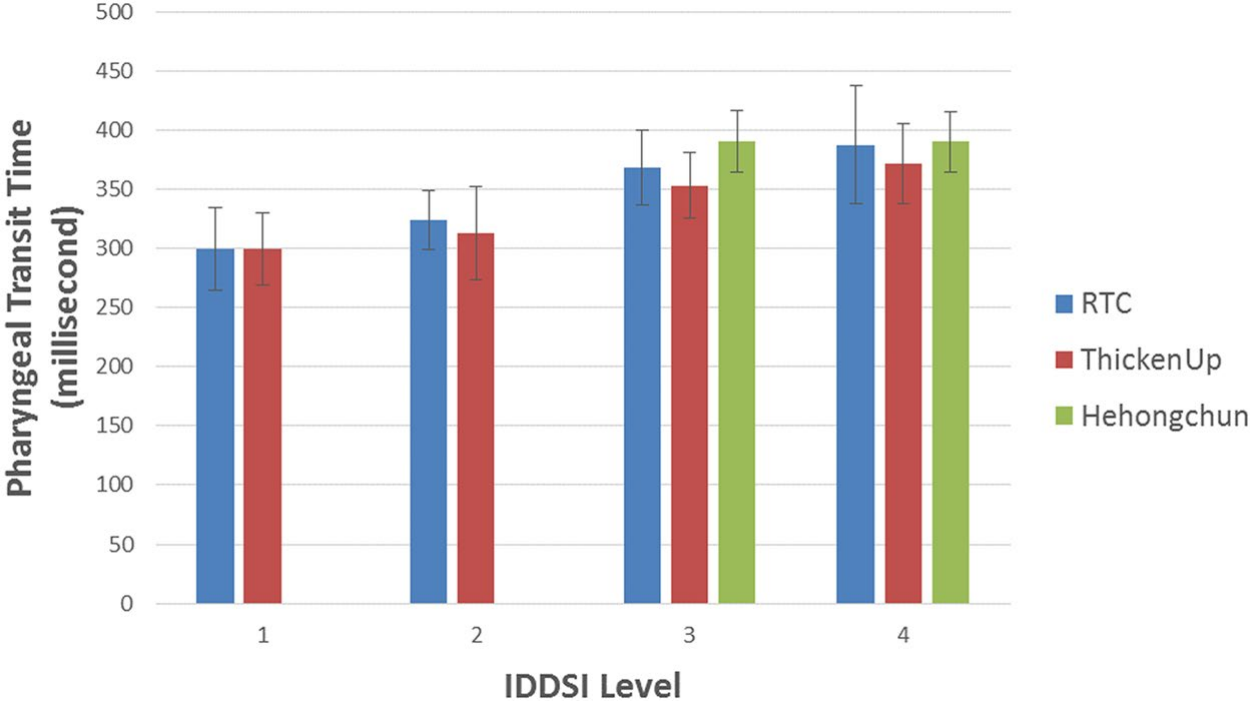
Pharyngeal transit time for all of the samples. Error bars are 95% confidence interval (n = 8).

Table 2 summarized the pharyngeal transit time and the bolus aspect ratio for all of the samples. It can be seen that the pharyngeal transit time generally increased with thicker samples (see Fig. 5). However, significant differences were only observed between samples that were thickened to IDDSI Level 1 and samples that were thickened to IDDSI Level 4 (p < 0.01). Additionally, it was observed that there was no statistically significant difference in pharyngeal transit time between samples with high and low extensional viscosity (p > 0.53) and between samples with high and low shear viscosity (p > 0.42).

**Table 2:** Summary of the pharyngeal transit time and bolus aspect ratio of the samples (±95% confidence interval).

Sample No.	Thickener	Thickener Concentration %	Pharyngeal Transit Time (ms)*	Bolus aspect ratio*
1	RTC	0.71	299 ± 35^b^	6.81 ± 0.43^ab^
2	ThickenUp	3.62	300 ± 31^b^	7.14 ± 0.43^a^
3	RTC	1.18	324 ± 25^ab^	6.71 ± 0.37^ab^
4	ThickenUp	4.35	313 ± 39^ab^	6.69 ± 0.38^ab^
5	RTC	2.61	368 ± 32^ab^	5.79 ± 0.36^cd^
6	ThickenUp	4.86	353 ± 27^ab^	6.23 ± 0.39^bc^
7	RTC	5.60	387 ± 50^a^	5.55 ± 0.42^d^
8	ThickenUp	5.34	372 ± 34^a^	6.50 ± 0.45^bc^
9	Hehongchun	4.97	391 ± 26^a^	5.86 ± 0.46^cd^
10	Hehongchun	9.21	390 ± 26^a^	5.69 ± 0.35^d^

Note: *Mean values in the same column that do not share a letter are statistically different (p < 0.05).

**Figure 6:**
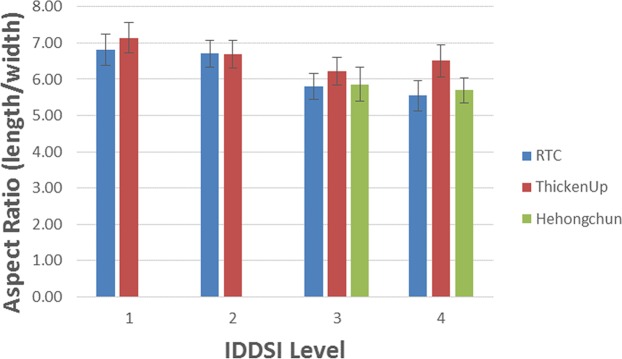
Bolus aspect ratio (length/width) for all of the samples. Error bars are 95% confidence interval (n = 8).

On the other hand, it was observed that the bolus aspect ratio for high extensional viscosity fluid was generally lower than for low extensional viscosity fluid (p < 0.01) (see Fig. 6). This shows that the high extensional viscosity samples were less elongated than low extensional viscosity samples. This becomes more evident for boluses at higher IDDSI Levels of consistency (IDDSI Level 4). However, there were no statistically significant differences in bolus aspect ratio as a function of shear viscosity (p > 0.38) (i.e. between RTC and Hehongchun thickeners).

Pharyngeal coating post swallow was noted on 61 number of samples across all participants. A slight residual coating was observed in the pharyngeal area of the participants after swallowing total of 51 ThickenUp samples and 10 Hehongchun samples across all participants.

## Discussion

The aims of this study were to (a) observe the effect of extensional as well as shear viscosity on pharyngeal transit time and the elongation of the bolus during swallowing, (b) evaluate the flow behavior of thickened fluids during swallowing through Videofluoroscopy Swallow Study (VFSS). It was observed that the pharyngeal transit time increased as the samples were thickened to higher IDDSI Levels. However, it was only statistically different between samples that were thickened to IDDSI Level 1 and IDDSI Level 4. These results are consistent with reports for longer pharyngeal transit time with a semi-solid bolus as compared with a liquid bolus in healthy adults^31,32^. It is hypothesized that the swallowing mechanism for healthy individuals are automatically adjusted, so that the bolus can safely flow through the pharynx during swallowing. For example, the tongue pressure generated by healthy individuals increases when they are swallowing thicker boluses^33,34^. Perhaps the difference in pharyngeal transit time between different IDDSI Levels is more prominent if swallowed by individuals with dysphagia. Clinical studies would be required to test this hypothesis.

The results from this study showed that fluids with a higher extensional viscosity reduce the elongation of the bolus during swallowing (sample 5 & 6; sample 7 & 8). This shows that the bolus has the tensile strength to hold together and resist breakage as the bolus passes through pharynx during swallowing. Extensional rheology testing tests the tensile strength as the sample is stretched linearly. During swallowing, the bolus is propelled by the tongue but also subject to external forces on the outside of the sample as the bolus flows through the pharynx in addition to gravity. Although extensional viscosity tests the sample in a linear stretching fashion, it would appear that it provides a proxy measure for bolus elongation during swallowing in healthy individuals. Further studies are needed with individuals with dysphagia where there are difference in lingual propulsion and pressure on the bolus during swallowing to determine whether tensile strength as tested using extensional viscosity is able to predict the thickener characteristics that result in boluses that elongate without breaking at difference thickness levels.

The results of the current study add to the literature by demonstrating that extensional viscosity is as important to consider as the traditional shear viscosity. When the fluids were mixed with RTC, the viscosity of the fluid is built through the development of a colloidal network, based on intermolecular hydrogen bonding among the xanthan gum molecules, in addition to limited polymer entanglement^35^. The presence of the polymer entanglement in the molecule is believed to be the reason of the enhanced extensional viscosity. On the other hand, ThickenUp is a swollen starch and partly dissolved which results in a liquid with some weak interlinking bonds between the granules and as such results in a highly heterogeneous fluid that has low extensional viscosity^21,23^. These results demonstrate that the molecular structure of the thickening agent has an impact on bolus cohesion and ability to resist fracture as it is stretched under gravity and external pressure on the bolus during the swallowing process.

It was observed that the bolus aspect ratio was similar for extensional controlled samples (sample 5 & 9; sample 7 & 10), but where shear viscosity varied. These results demonstrate that the extension characteristics were better able to predict shorter elongation of the bolus during swallowing that the shear viscosity characteristics. The higher maximum extensional viscosity predicts that the bolus has better tensile strength to be able to hold together, despite physiological attempts at deformation during bolus propulsion through the oropharynx. Tensile strength that resists breakage during deformation and elongation^36^ could potentially be considered a proxy measure by the clinician for cohesion. The ability of the bolus to resist breakage during swallowing is an important bolus parameter for individuals with dysphagia as a bolus that is less cohesive tends to fracture into multiple boluses during swallowing and may get caught in the pharynx as residue^18^. In the current study, when the bolus was elongated during healthy swallowing, highly cohesive fluids (boluses with high extensional viscosity) were able to retain their shape as a bolus (remain as one contiguous bolus) and flow through the pharyngeal area without breaking.

**Figure 7:**
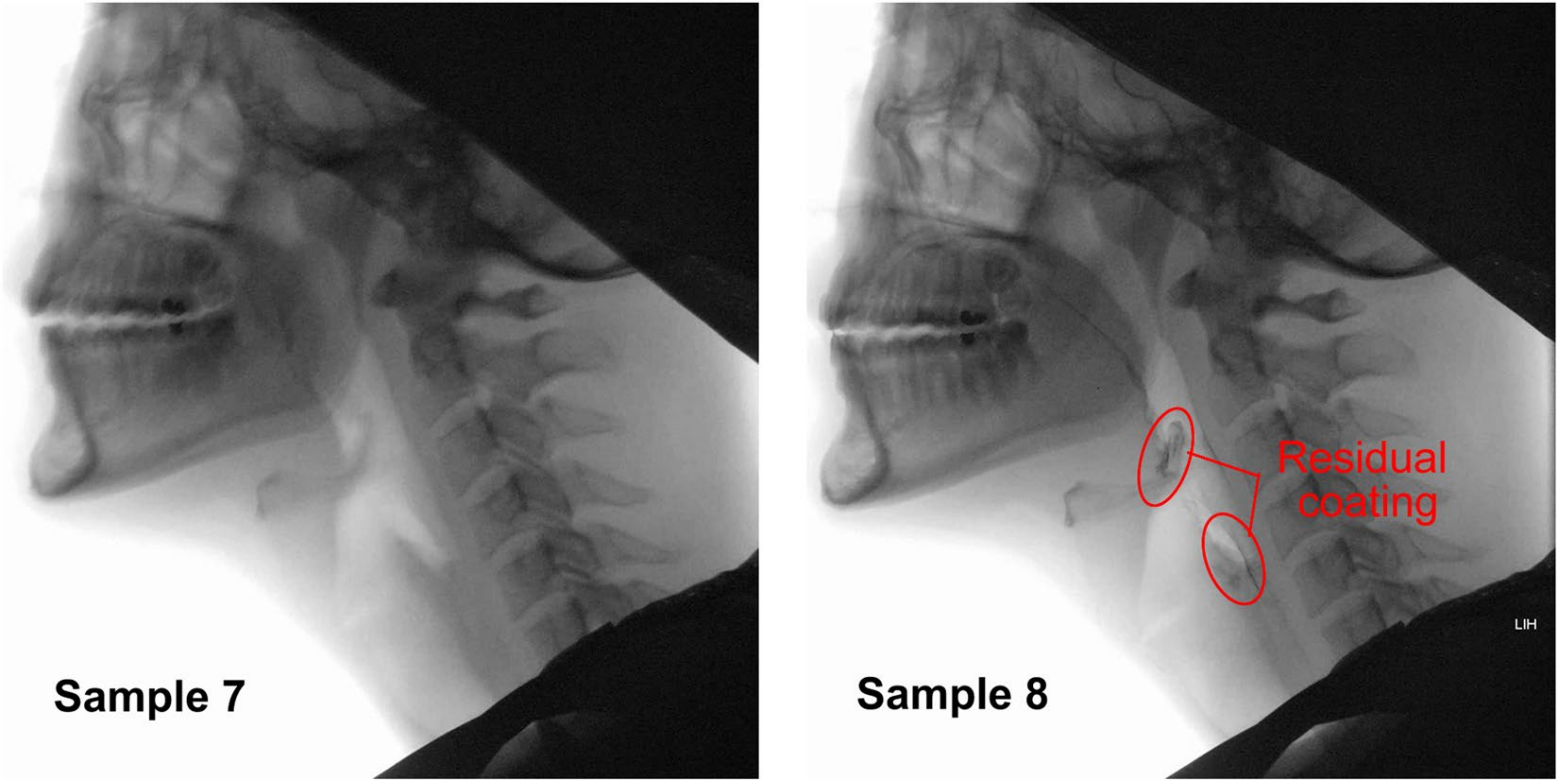
Photographic of residual coating left in the pharyngeal area after swallowing for sample 7 (RTC 5.60%, no residue) and sample 8 (ThickenUp 5.34%, some residual coating), having equal apparent viscosity at 50 s^−1^.

In regards to an evaluation of flow behavior of thickened fluids during swallowing as observed during VFSS, a slight coating was observed in the pharyngeal area of the participants after swallowing low extensional viscosity samples (see Fig. 7). It should be noted that healthy individuals typically do not have pharyngeal residue when they swallow thin fluids like water. However, thin coatings of residue could be observed when swallowing fluids that are slightly adhesive, such as starch thickened fluids. It is hypothesized that the residual coating was visible only after swallowing starch based thickener, because this type of thickened liquids is perceived as adhesive and sticky, as opposed to gum based thickeners that are perceived as slippery^37^.

In addition to textural properties such as adhesiveness and slipperiness, barium concentration has previously been identified as a confounding element for pharyngeal residue as noted by Steele *et al*.^38^. Steele *et al*.^38^ reported that barium concentration of 60% w/v or higher may leave a visible thin line of residual coating along both the pharyngeal and esophageal mucosa. As mentioned previously, 60% w/v barium concentration was also used in this study. It can be seen from Fig. 7, the coating residue observed from this study was a thin line of barium on the base of tongue, extending to the valleculae and piriform sinuses area. However, all of the samples in this study were mixed with the same barium concentration. Therefore, it is hypothesised that the residue observed in this study was due to different rheological or microstructural properties of the bolus (i.e. low extensional viscosity coupled with non-homogenous microstructure) as found in the starch based thickeners. When the links between molecules at a microstructural level are weak, it is logical that this might result in low extensional viscosity and therefore poor tensile strength under stretching, increasing the likelihood of bolus breakage. The non-homogenous microstructure also allows other textural features such as adhesiveness to play a more dominant role. It has been reported that starch based thickeners are perceived as more adhesive than gum based thickeners^37^. A sample with a combination of poor tensile strength coupled with adhesive qualities may explain the bolus fracture and tendency to stick as a light coating of residue with the starch based samples seen in this study. Clinicians should be aware that starch based thickened liquids as assessed in this study presented with low extensional viscosity and may be more inclined to fracture. These results are especially important when interpreting VFSS studies from people with dysphagia. Understanding the microstructural and tensile strength properties of the test substance may have implications for the qualities of the thick liquid that best meets the person’s swallowing safety needs.

The phenomenon associated with sample breakage as associated with sample microstructure had been previously reported by Vilardell *et al*.^39^ and explored with dysphagic patients. It was observed that xanthan gum thickener did not increase the prevalence of pharyngeal residue, while modified starch thickener increased pharyngeal residue in spite of the samples being of the similar shear viscosity. As noted above and confirmed by other research, this is most likely because the extensional viscosity of the modified starch thickened sample was lower than the xanthan gum thickened samples^21,22^.

It should be noted that the current study was conducted only with healthy individuals, but it is expected that the consequence and impact of variation in samples of varying extensional rheology will be more evident should the experiments be conducted with individuals with dysphagia. In addition, the range of extensional viscosity variation in this study is limited due to the rheological nature of the biopolymers. However, at the levels of extensional viscosity assessed in this study, the evidence was clear enough to observe an effect of extensional viscosity of the bolus on swallowing that is different to usually reported effects of shear viscosity.

## Conclusions

Videofluoroscopy Swallow Study (VFSS) was used to evaluate the flow behavior of thickened fluids during swallowing. The relevance of shear and extensional viscosity of the bolus in swallowing were investigated in this study. The pharyngeal transit time generally increases when the fluids are thickened to higher IDDSI drink thickness levels. A very important observation in this study is that, for boluses of similar shear viscosity, increasing the maximum extensional viscosity reduces the elongation of the bolus during swallowing. Our results further suggest that the maximum extensional viscosity is related to the tensile strength of the bolus and ability to resist stretching and deformation, as determined by the thickener microstructure. Starch and gum based thickeners have different microstructures resulting in different extensional rheology even if shear viscosity is matched. Higher maximum extensional viscosity leads to bolus that is stronger and more resistant to elongation^21^. Therefore, the bolus was able to retain its shape as a bolus and flow through the pharyngeal area without fracturing when elongated during swallowing.

It was observed that residual coating was visible only after swallowing starch based thickener. It is hypothesised that this is because of low extensional viscosity as found in the starch based thickener and that the non-homogeneous and sticky nature of the swollen starch granules allowed the bolus to fracture and then stick to the mucosa of the pharynx^37^. Future studies are required to observe the significance of textural properties such as bolus’ stickiness, especially when couple with poor tensile strength during deformation swallowing. Future clinical studies are required to evaluate the flow behavior of thickened fluids that vary according to shear and extensional rheology during swallowing by individuals with dysphagia.
